# Evaluation einer SARS-CoV-2-Teststrategie zu Beginn der COVID-19-Pandemie in einem südwestdeutschen Universitätsklinikum

**DOI:** 10.1007/s00103-021-03287-z

**Published:** 2021-02-12

**Authors:** Anna Trimborn, Marlis Gerigk, Alexandra Heininger, Nandhini Santhanam, Thomas Walter, Bettina Lange

**Affiliations:** 1 Medizinische Fakultät Mannheim, Universitätsmedizin Mannheim, Stabsstelle Krankenhaushygiene, Universität Heidelberg, Theodor-Kutzer-Ufer 1–3, 68167 Mannheim, Deutschland; 2 Medizinische Fakultät Mannheim, Universitätsmedizin Mannheim, Institut für Medizinische Mikrobiologie und Hygiene, Universität Heidelberg, Theodor-Kutzer-Ufer 1–3, 68167 Mannheim, Deutschland; 3 Medizinische Fakultät Mannheim, Universitätsmedizin Mannheim, Heinrich Lanz-Zentrum für digitale Gesundheit, Universität Heidelberg, Theodor-Kutzer-Ufer 1–3, 68167 Mannheim, Deutschland; 4 Medizinische Fakultät Mannheim, Universitätsmedizin Mannheim, Zentrale Notaufnahme, Universität Heidelberg, Theodor-Kutzer-Ufer 1–3, 68167 Mannheim, Deutschland

**Keywords:** Orientierungshilfe, Testkriterien, Testkapazitäten, Robert Koch-Institut, Geovisualisierung, Guideline, Testing criterias, Testing capacities, Robert Koch Institute, Geovisualisation

## Abstract

**Hintergrund:**

Zu Beginn der COVID-19-Pandemie in Deutschland veröffentlichte das Robert Koch-Institut (RKI) Orientierungshilfen für eine bedarfsadaptierte und zielgerichtete Teststrategie auf SARS-CoV‑2. In der Universitätsmedizin Mannheim (UMM) wurde von Beginn an eine RKI-konforme Teststrategie, basierend auf den RKI-Orientierungshilfen angewendet. Ziel dieser Arbeit ist es, zu klären, ob sich diese RKI-konforme Teststrategie als lohnenswert erwiesen hat.

**Material und Methoden:**

Es erfolgte eine retrospektive Auswertung aller SARS-CoV-2-Untersuchungen im Zeitraum 26.02.–06.04.2020. Mithilfe der Adressinformationen der getesteten Personen erfolgte zudem eine Geovisualisierung der SARS-CoV-2-Testhäufigkeiten und der positiven Testergebnisse im Stadtgebiet Mannheim.

**Ergebnisse:**

Der Anteil SARS-CoV-2-positiver Befunde bei den diagnostischen Proben (*n* = 2808) lag durchschnittlich bei 7 %. Ein Mitarbeiterscreening (*n* = 448) blieb ohne positiven Befund. Es erfolgte lediglich ein nosokomialer SARS-CoV-2-Nachweis, Ausbruchsgeschehen gab es nicht. Die Geovisualisierung zeigte innerhalb des Untersuchungszeitraumes eine Verschiebung der Testhäufigkeiten und der lokalen Häufigkeit positiver SARS-CoV-2-Befunde im Stadtgebiet Mannheim.

**Diskussion:**

Die festgelegte RKI-konforme Teststrategie führte sowohl zu einem stabilen Anteil positiver Ergebnisse als auch zu einer bedarfsadaptierten Anpassung der Testkapazitäten. Dadurch hat sich diese Strategie unter Praxisbedingungen aus infektionshygienischer und -präventiver Sicht als effektiv erwiesen. Geovisualisierungen können dabei helfen, innerhalb eines Stadtgebietes Verschiebungen von Infektionsherden deutlich zu machen, was im Sinne gezielter infektionspräventiver Maßnahmen (z. B. Impfkampagnen) sinnvoll eingesetzt werden kann.

## Abstract (en)

**Background:**

At the beginning of the COVID-19 pandemic, the German Robert Koch Institute (RKI) published several guidelines addressing the medical health services helping to detect SARS CoV‑2. Needing an available and specific test strategy regarding SARS-CoV‑2, our own test strategy strictly followed these testing criteria.

**Materials and methods:**

Using a retrospective analysis, we verified if such a test strategy was an effective tool in the context of infection prevention control and as reliable SARS-CoV‑2 detection. Therefore, we analysed our own test results of suspected SARS-CoV‑2 cases between 26 February and 6 April 2020. Additionally, we used a geovisualisation tool to visualise test frequencies and positive test results within different districts of Mannheim based on people’s addresses.

**Results:**

There were on average 7% positive test results of SARS-CoV‑2 within a population with typical symptoms of COVID-19 (*n* = 2808). There was no positive test result within an asymptomatic population (*n* = 448). However, one positive test result turned out to be a nosocomial infection. Finally, geovisualisation highlighted a shift of test frequencies and local positive rates for SARS-CoV‑2 from one district of Mannheim to another.

**Discussion:**

In conclusion, our test strategy strictly based on testing criteria suggested by the Robert Koch Institute resulted in a steady rate of positive tests and allowed us to increase test capacity without causing numbers of nosocomial infections of COVID-19. Geovisualisation tools can offer support in analysing an ongoing spread of transmissible diseases. In the future, they could be used as helpful tools for infection prevention control, for example in the context of vaccination programs.

## Einleitung

Im Januar 2020 wurde in ersten Publikationen über ein neuartiges Coronavirus (SARS-CoV-2) als Auslöser einer überwiegend durch Tröpfcheninfektion übertragenen respiratorischen Erkrankung (COVID-19) berichtet [1]. Am 11.03.2020 erklärte die Weltgesundheitsorganisation (WHO) COVID-19 bei weltweit zunehmenden Fallzahlen zur Pandemie [2]. Zur gezielten Detektion von COVID-19-Erkrankten publizierte das Robert Koch-Institut (RKI) zu Beginn der Pandemie im Zeitraum vom 26.02.2020 bis 06.04.2020 4 aufeinanderfolgende Flussschemata als Orientierungshilfe für eine bedarfsadaptierte Teststrategie auf SARS-CoV‑2 [3]. Alle 4 Versionen beruhten auf einer Kombination aus klinischen und epidemiologischen Kriterien, aufgrund derer die Indikation für eine Testung zu prüfen war [4]. Dabei wurde zwischen begründeten Verdachtsfällen und Fällen unter differenzialdiagnostischer Abklärung unterschieden. Eine sofortige Testung war angesichts knapper Ressourcen nur bei Erfüllung der Kriterien des begründeten Verdachtsfalles indiziert.

In der Universitätsmedizin Mannheim (UMM) wurde von Beginn an eine RKI-konforme Teststrategie, basierend auf der jeweiligen RKI-Orientierungshilfe angewendet und bis zum 06.04.2020 strikt beibehalten. Die empfohlenen klinisch-epidemiologischen Prüfkriterien wurden jeweils vollständig in die klinikspezifischen Verfahrensanweisungen übernommen. Eine sofortige Testung auf SARS-CoV‑2 erfolgte ausschließlich gemäß den geltenden Empfehlungen des RKI. Im Stadtgebiet Mannheim (mit ca. 350.000 Einwohnern) wurden in dieser Zeit etwa 75 % der gesamten SARS-CoV-2-Diagnostik durch das Universitätsklinikum Mannheim durchgeführt. Von Beginn der Pandemie bis zum 06.04.2020 wurden im Stadtgebiet Mannheim 302 COVID-19-Fälle registriert, was einer Inzidenz von 97,7 pro 100.000 Einwohner in diesem Zeitraum entspricht [5].

Ziel dieser retrospektiven Analyse ist es, anhand der eigenen Testergebnisse die Praktikabilität und Wirksamkeit einer RKI-konformen Teststrategie im Rahmen der COVID-19-Pandemie an einem Universitätsklinikum zu evaluieren und zu diskutieren.

## Methodik

Anhand der sequenziellen Versionen der RKI-Orientierungshilfen, angepasst an die epidemiologische Lage, wurde der Zeitraum der eigenen Analyse in 3 Abschnitte gegliedert: Abschnitt 1 vom 26.02.2020 bis 12.03.2020, Abschnitt 2 vom 13.03.2020 bis 24.03.2020 und Abschnitt 3 vom 25.03.2020 bis 06.04.2020.

Das Material für die SARS-CoV-2-Untersuchungen wurde gemäß RKI-Empfehlung durch einen gepoolten oro- und nasopharyngealen Abstrich gewonnen [6]. Nur bei ausgewählten stationären Patientinnen und Patienten wurde zusätzlich eine Probe aus den tiefen Atemwegen untersucht. Die Proben wurden durch das Institut für Medizinische Mikrobiologie und Hygiene (IMMH) der UMM bearbeitet. Die retrospektive Analyse der Ergebnisse erfolgte durch eine SQL-Abfrage des Laborinformationssystems i/med® der Firma DORNER® (DORNER GmbH & Co. KG, Mühlheim, Deutschland). Die Testung auf SARS-CoV‑2 erfolgte mittels RT-Polymerase-Kettenreaktion (PCR): Bis zum 19.03.2020 wurden ausschließlich kommerzielle SARS-CoV-2-Testkits der Firma TIB MOLBIOL® (TIB Molbiol, Berlin, Deutschland), ab dem 19.03.2020 zusätzlich von der Firma Altona Diagnostics® (altona Diagnostics GmbH, Hamburg, Deutschland) verwendet.

Eingeschlossen in die Datenanalyse wurden alle Ergebnisse der in der UMM durchgeführten Untersuchungen inklusive der Screeningergebnisse externer Altenheimmitarbeiterinnen und -mitarbeiter. Ausgeschlossen wurden Ergebnisse präinterventioneller Screeninguntersuchungen innerhalb der UMM sowie Ergebnisse von Mitarbeitertestungen bei Kontaktermittlungen. Für die Datenanalyse wurden die Befunde jeder getesteten Person im jeweiligen Beobachtungszeitraum nur einmal gewertet, im Falle einer mehrfachen Untersuchung derselben Person wurde bei diskordanten Befunden das positive Ergebnis berücksichtigt.

Im Beobachtungszeitraum wurden insgesamt 2808 diagnostische Untersuchungen von symptomatischen Personen gemäß RKI-Kriterien auf SARS-CoV‑2 durchgeführt [4]. Diese wurden initial zu 95 % und im letzten Drittel des Untersuchungszeitraumes zu 90 % aus ambulant getesteten Patientinnen und Patienten rekrutiert. Die verbleibenden 5 % bzw. 10 % der untersuchten Personen bildeten stationäre Patientinnen und Patienten mit COVID-19-verdächtiger Symptomatik (Tab. 1). Ein Screening aller stationär aufgenommenen Patientinnen und Patienten wurde nicht durchgeführt. Zusätzlich zu diesen diagnostischen Proben wurden im Untersuchungszeitraum 448 Screeningproben von asymptomatischen Mitarbeiterinnen und Mitarbeitern von Altenheimen im Stadtgebiet Mannheim untersucht. Zum Zeitpunkt der Screeninguntersuchung erfüllte keine Mitarbeiterin und kein Mitarbeiter die klinisch-epidemiologischen Kriterien des RKI, eine Untersuchung erfolgte ausschließlich aufgrund der Tätigkeit in einem vulnerablen Bereich (Altenheim). Einmalig wurde zudem anlassbezogen ein Screening aller Patientinnen und Patienten einer Station durchgeführt, nachdem zwei Patienten dort zeitgleich je 5 Tage nach Aufnahme COVID-19-typische Symptome und ein positives Testergebnis gezeigt hatten.

**Table 1:** 

Untersuchungsabschnitt	Medianes Alter (Jahre)	Geschlecht (männlich, %)	Abnahmesetting (stationär, %)	Ranking der Bezirke nach Zahl der Tests pro 100.000 Einwohner
*1* *(26.02.–12.03.2020)*	44 (Range: 1–87)	52	5	1. Käfertal 2. Wallstadt 3. Neuostheim
*2* *(13.03.–24.03.2020)*	47,5 (Range: 0–95 Jahre)	47	5	1. Neuostheim 2. Gartenstadt 3. Feudenheim
*3* *(25.03.–06.04.2020)*	48,5 (Range: 0–97 Jahre)	46	10	1. Neuostheim 2. Friedrichsfeld 3. Gartenstadt

Für die Geovisualisierung wurden die Adressinformationen (Postleitzahl) der getesteten Personen dem Krankenhausinformationssystem SAP ISH® (SAP SE®, Walldorf, Deutschland) entnommen, die SARS-CoV-2-Testergebnisse wurden durch i/med® ermitteltet. Die Angaben über die Einwohnerzahlen der Stadtbezirke stammen aus dem Open-Data-Portal der Stadt Mannheim [7]. In Zusammenschau erfolgte jeweils eine Geovisualisierung der SARS-CoV-2-Testhäufigkeiten und der positiven Testergebnisse bezogen auf die Einwohnerzahlen der jeweiligen Stadtbezirke mithilfe des Leaflet-Packages [8] der Statistiksoftware R [9] und Kartenmaterials der OpenStreetMap [10].

## Ergebnisse

Im gesamten Untersuchungszeitraum wurden im Institut für Medizinische Mikrobiologie und Hygiene (IMMH) 2808 diagnostische Proben auf SARS-CoV‑2 untersucht. Die Kohorte bestand zu 47 % aus männlichen Personen, das mediane Alter betrug 48,5 Jahre (Range: 0–97 Jahre). Insgesamt 205 (7 %) dieser Proben zeigten ein positives Testergebnis. Die demografischen Daten für die jeweiligen Untersuchungsabschnitte können der Tab. 1 entnommen werden.

**Figure 1:**
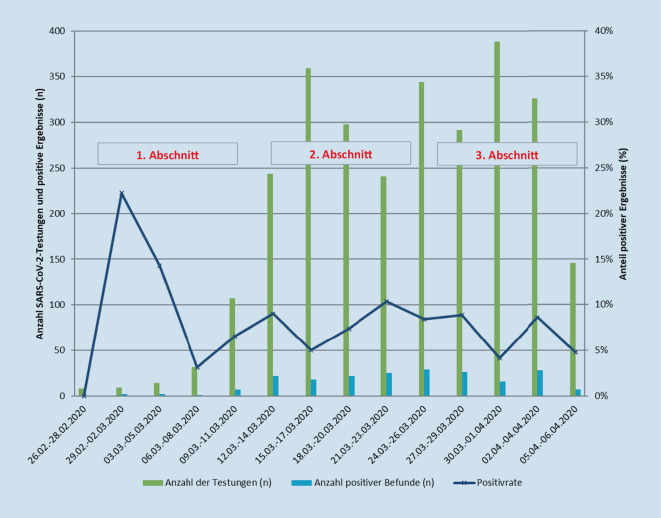

Im ersten Beobachtungsabschnitt wurden 228 Untersuchungen auf SARS-CoV‑2 durchgeführt, von diesen waren 14 (6 %) Testergebnisse positiv (Abb. 1: blaue Balken); 95 % (216/228) der Untersuchungen erfolgten im ambulanten Setting. Im zweiten Beobachtungsabschnitt lag die Zahl der Untersuchungen bei insgesamt 1181 (Abb. 1: grüne Balken), von diesen erfolgten 1125 (95 %) im ambulanten Setting. Der Anteil positiver Ergebnisse in der Betrachtung von je 3 Tagen lag in diesem Untersuchungszeitraum zwischen minimal 5 % und maximal 10 % (Positivrate, in Abb. 1: blaue durchgehende Linie); die mittlere Positivrate betrug 8 % (Abb. 1). Im letzten Beobachtungsabschnitt wurden 1399 Testungen mit insgesamt 98 positiven Befunden (7 %; minimale/maximale Positivrate 4 bzw. 9 %) durchgeführt (Abb. 1). Der Anteil durchgeführter Untersuchungen im stationären Setting verdoppelte sich und stieg damit auf 10 % (Tab. 1).

Bei der Untersuchung von 448 asymptomatischen Mitarbeiterinnen und Mitarbeitern von Mannheimer Altenheimen auf SARS-CoV‑2 konnte im Untersuchungszeitraum kein positives Ergebnis festgestellt werden.

Im gesamten Untersuchungszeitraum wurde in der UMM nur ein Fall einer asymptomatischen, vermutlich nosokomialen SARS-CoV-2-Infektion (Nachweis am Tag 16 nach Aufnahme; weitere zeitliche Zuordnung bei Fehlen von Symptomen nicht möglich) im Rahmen eines Stationsscreenings nachgewiesen; weitere nosokomiale Fälle wurden nicht beobachtet.

**Figure 2:**
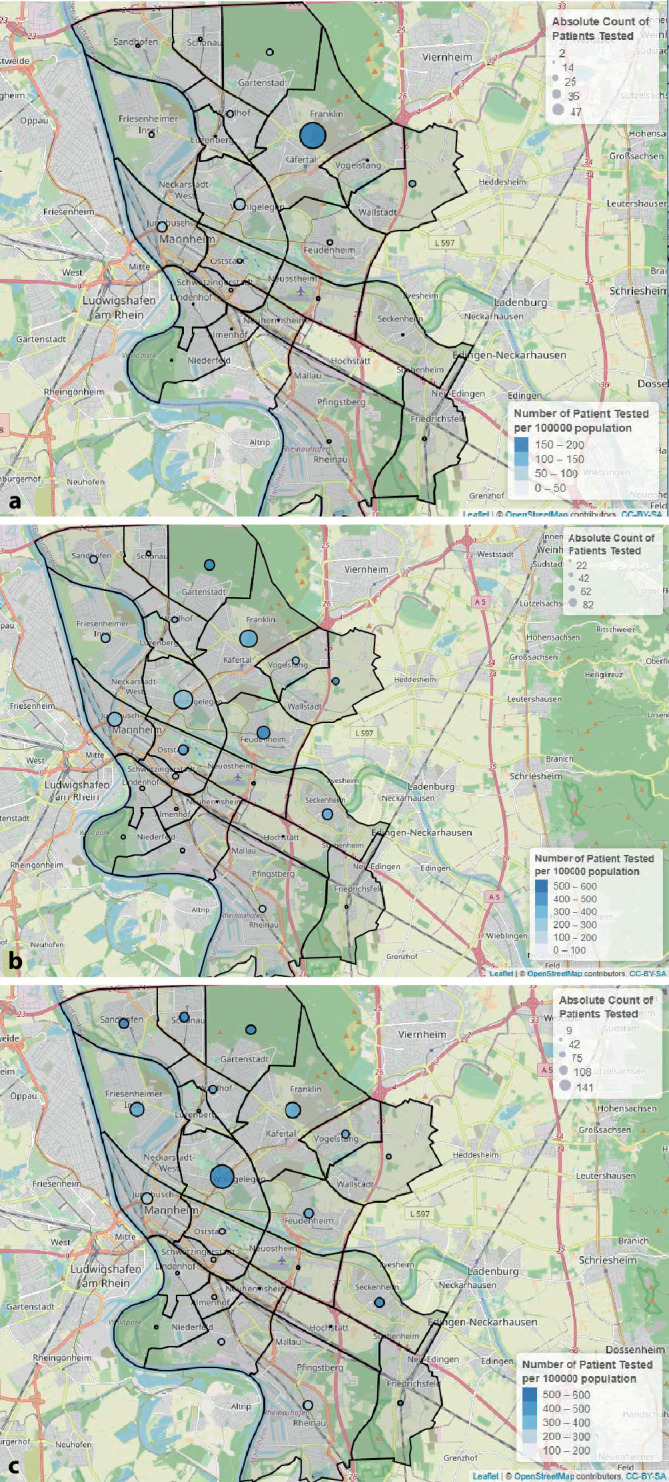

**Figure 3:**
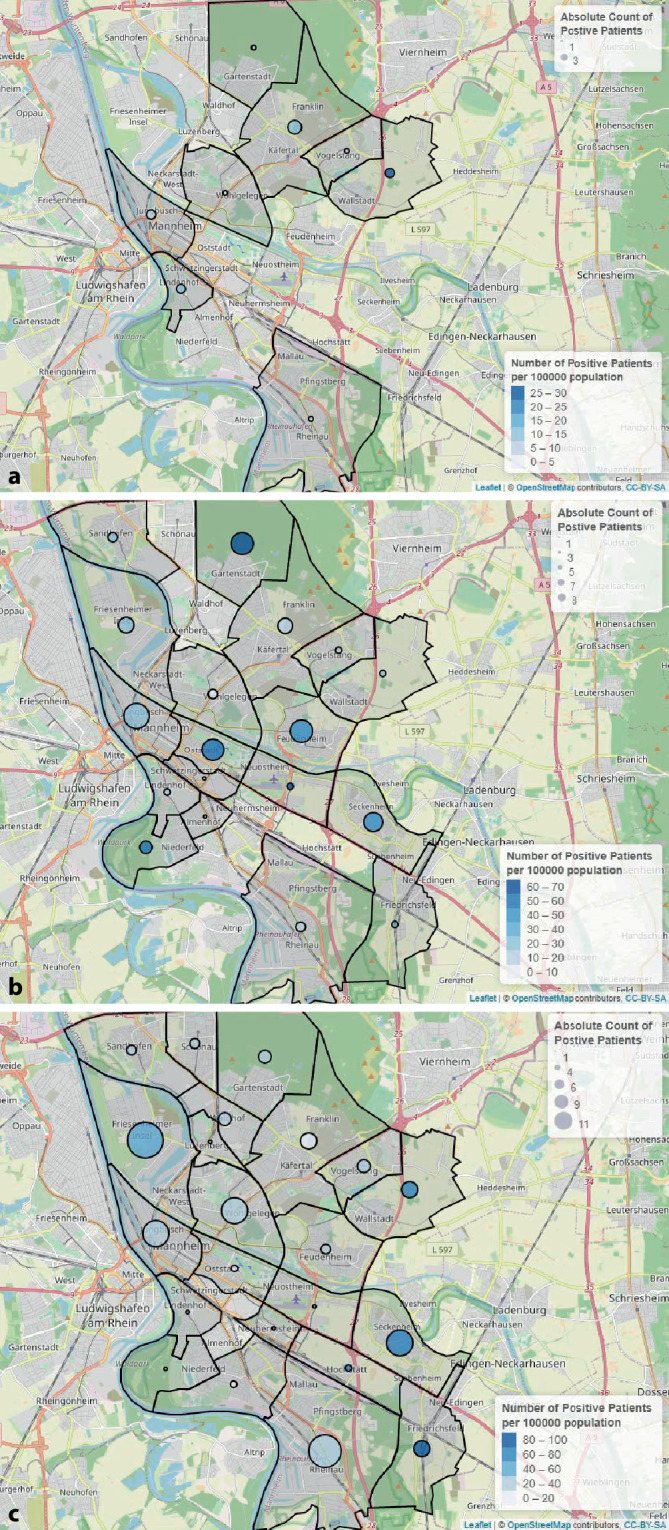

Die räumliche Verteilung der Anzahl von SARS-CoV-2-Untersuchungen innerhalb des Stadtgebiets Mannheim und innerhalb der jeweiligen Analyseabschnitte ist detailliert der Abb. 2 zu entnehmen. Die lokale Häufigkeit positiver Befunde (pro 100.000 Einwohner) variierte innerhalb der 3 Untersuchungsabschnitte deutlich und zeigte eine Verschiebung innerhalb der Stadtgebiete (Abb. 3).

## Diskussion

Die Ergebnisse dieser Untersuchung zeigen, dass sich eine streng RKI-konforme Teststrategie in den Anfangswochen der COVID-19-Pandemie unter den Praxisbedingungen eines Universitätsklinikums aus infektionshygienischer und -präventiver Sicht als effektiv erwiesen hat.

Der Anteil positiver SARS-CoV-2-Testergebnisse im Einzugsgebiet der UMM stimmt mit durchschnittlich 7 % (Abb. 1) mit den bereits publizierten bundesweiten Ergebnissen des RKI gut überein [11]. Im Vergleich aller 3 Untersuchungsabschnitte zeigte sich der Prozentsatz positiver Ergebnisse zudem weitgehend stabil. Das RKI-konforme Vorgehen darf daher als effektiv bewertet werden, weil hierdurch eine Balance zwischen nur eingeschränkt verfügbaren Testressourcen und der Vermeidung nosokomialer Infektionen gelungen ist. Demgegenüber zeigte eine Screeninguntersuchung in einer chinesischen Fieberklinik nahe der Hochinzidenzregion Hubei eine Positivrate von nur 1,3 % [12]. Diese Gegenüberstellung belegt, dass die Empfehlungen des RKI eine zielgerichtete, ressourcensparende Steuerung ermöglichten.

Die inhaltlichen Veränderungen der RKI-Empfehlungen orientierten sich an der tatsächlichen epidemiologischen Situation: Vor Beginn der epidemischen Phase in Deutschland handelte es sich um einzelne, nicht zusammenhängende COVID-19-Infektionscluster [13]. Ab Anfang März 2020 entwickelte sich ein deutschlandweites Ausbruchsgeschehen mit deutlich zunehmenden Fallzahlen, analog zu den Entwicklungen in anderen europäischen Ländern [13, 14]. Diese Entwicklung wurde abgebildet durch eine Veränderung der Testkriterien, die initial auf Reiserückkehrer aus Risikogebieten ausgerichtet waren und die sich im Weiteren stärker auf die individuelle Symptomatik und die epidemiologische Situation im engeren Umfeld der Personen fokussierten [4]. Die stetige Anpassung der RKI-Testkriterien erlaubte es der UMM, diesem entscheidenden Übergang mit einer bedarfsgerechten Steigerung der Testkapazitäten zu begegnen. Die Verschiebung des inhaltlichen Fokus der Testkriterien führte im dritten Untersuchungsabschnitt zu einer Verdopplung des Anteils im stationären Setting getesteter Personen. Dadurch wurden Fälle, die wegen anderer Grunderkrankungen zur stationären Behandlung aufgenommen worden waren, mit hoher Sensitivität detektiert. Im Ergebnis konnte innerhalb der UMM die von Rieg et al. [15] beschriebene Gefahr einer erheblichen nosokomialen Ausbreitung von SARS-CoV‑2 beherrscht werden.

Die Ergebnisse, gewonnen einerseits durch die RKI-konforme Teststrategie und andererseits durch eine Screeninguntersuchung von Altenheimmitarbeiterinnen und -mitarbeitern, verdeutlichen, dass vergleichsweise sehr hohe Testkapazitäten aufgewendet werden müssen, um anhand eines Screenings gezielt infektionspräventive Maßnahmen ergreifen zu können. Im Rahmen mittlerweile stark erhöhter Testkapazitäten empfiehlt die bundesweite Teststrategie des Bundesministeriums für Gesundheit eine kombinierte Vorgehensweise aus Screening und gezielter Testung [16].

Durch die ergänzende Geovisualisierung der Daten im Stadtgebiet Mannheim wird deutlich, dass sich in den einzelnen Abschnitten des Beobachtungszeitraumes die Positivraten in den Stadtbezirken verändert haben. Bezirke mit initial niedriger Positivrate zeigten im Beobachtungsverlauf eine Zunahme der Infektionsraten, gleichzeitig erfolgte eine Verschiebung der Positivraten innerhalb der Stadtbezirke. Die über die 3 Untersuchungsabschnitte hinweg relativ konstante Rate positiver SARS-CoV-2-Testergebnisse spricht dafür, dass interferierende Effekte durch Unterschiede in den betroffenen Subpopulationen ausreichend ausgeglichen werden konnten. Unabhängig davon sollten die Verschiebungen der stärker betroffenen Stadtgebiete vor einer möglichen zweiten COVID-19-Infektionswelle berücksichtigt werden. Die besondere Relevanz einer Geovisualisierung mit hoher Auflösung innerhalb eines Stadtgebietes als Grundlage für gesellschaftsgesundheitliche Präventionsmaßnahmen gegen nichtübertragbare Erkrankungen zeigten bereits Noble et al. [17]. Die hier demonstrierten Ergebnisse weisen auf einen Nutzen der Geovisualisierung für die Infektionsprävention hin.

Eine Limitation dieser Untersuchung liegt in der nur mittleren COVID-19-Inzidenz im Stadtgebiet Mannheim, was den Vergleich mit anderen südwestdeutschen Universitätsstädten erschwert [5, 15]. Ferner bleibt zu erwähnen, dass die Untersuchungen auf SARS-CoV‑2 durch stetig wechselnde Untersucher durchgeführt wurden. Diese Überlegung erscheint jedoch nur theoretisch von Bedeutung, da alle Probenehmer vor Einsatz in der adäquaten Technik geschult wurden [18]. Darüber hinaus dürfte dieser Umstand die klinische Routine in nahezu allen deutschen Krankenhäusern abbilden. Aufgrund des retrospektiven Charakters ist weiterhin eine Abgrenzung laut RKI-Nomenklatur zwischen begründeten Verdachtsfällen und Fällen unter differenzialdiagnostischer Abklärung nicht möglich.

## Fazit

Zusammenfassend machen die Ergebnisse dieser Arbeit deutlich, dass die in der Anfangszeit der COVID-19-Pandemie für die UMM festgelegte RKI-konforme Teststrategie nicht nur zu einem stabilen Anteil positiver Ergebnisse, sondern auch zu einer bedarfsadaptierten Anpassung der Testkapazitäten geführt hat. Zukünftig können Geovisualisierungen dabei helfen, innerhalb eines Stadtgebietes Verschiebungen von Infektionsherden deutlich zu machen. Die Etablierung eines Datenintegrationszentrums über das im Rahmen der Medizininformatik-Initiative des Bundesministeriums für Bildung und Forschung (BMBF) geförderte MIRACUM-Konsortium (Medical Informatics in Research and Care in University Medicine; [19]) stellte eine wesentliche Voraussetzung für die rasche Umsetzung der beschriebenen Auswertungen und Geovisualisierungen dar. Die Erkenntnisse können im Sinne gezielter infektionspräventiver Maßnahmen (z. B. Impf- und Aufklärungskampagnen) zukünftig sinnvoll eingesetzt werden.
